# A Point Prevalence Survey of Healthcare-Associated Infections and Antimicrobial Use in Public Acute Care Hospitals in Crete, Greece

**DOI:** 10.3390/antibiotics11091258

**Published:** 2022-09-16

**Authors:** Petros Ioannou, Eirini Astrinaki, Efsevia Vitsaxaki, Emmanouil Bolikas, Despoina Christofaki, Apostolia Salvaraki, Eirini Lagoudaki, Eleni Ioannidou, Stamatis Karakonstantis, Stamatina Saplamidou, Christos Cleovoulou, Eleni Stamataki, Stavroula Ilia, Argyri Messaritaki, Michaela Avdi, Anthoula Chalkiadaki, Styliani Papathanasaki, Chrisanthi Markopoulou, Evagelia Magouli, Maria Moustaki, Vasileia-Athina Kataxaki, Panagiotis Skevakis, Nikolaos Spernovasilis, Georgios Chamilos, Diamantis P. Kofteridis

**Affiliations:** 1 Department of Internal Medicine, University Hospital of Heraklion, 71500 Heraklion, Greece; 2 Infection Control Committee, University Hospital of Heraklion, 71500 Heraklion, Greece; 3 Infection Control Committee, “Venizeleio-Pananeio” General Hospital of Heraklion, 71409 Heraklion, Greece; 4 Infection Control Committee, Organic Unit of Agios Nikolaos, General Hospital of Lasithi/General Hospital-Health Center of Neapoli “Dialinakio”, 72100 Agios Nikolaos, Greece; 5 Infection Control Committee, General Hospital of Rethymno, 74132 Rethymno, Greece; 6 Infection Control Committee, General Hospital of Chania “St. George”, 73300 Chania, Greece; 7 Pediatric Intensive Care Unit, University Hospital of Heraklion, 71500 Heraklion, Greece; 8 School of Medicine, University of Crete, 71500 Heraklion, Greece; 9 Office of Nursing Services, University Hospital of Heraklion, 71500 Heraklion, Greece; 10 Internal Medicine, General Hospital of Rethymno, 74132 Rethymno, Greece; 11 Infection Control Committee, Decentralized Organic Unit of Sitia, General Hospital of Lasithi/General Hospital-Health Center of Neapoli “Dialinakio”, 72300 Sitia, Greece; 12 Infection Control Committee, Decentralized Organic Unit of Ierapetra, General Hospital of Lasithi/General Hospital-Health Center of Neapoli “Dialinakio”, 72200 Ierapetra, Greece; 13 Infection Control Committee, General Hospital-Health Care Center of Neapoli “Dialinakeio”, 72400 Neapoli, Greece; 14 Collaborative Center for Clinical Epidemiology and Outcomes Research (CLEO), 15451 Athens, Greece; 15 German Oncology Center, 4108 Limassol, Cyprus; 16 Department of Clinical Microbiology, University Hospital of Heraklion, 71500 Heraklion, Greece

**Keywords:** infection control, point prevalence survey, antimicrobial stewardship, COVID-19, hospital acquired infection, antimicrobial resistance

## Abstract

Background: Both healthcare-associated infections (HAIs) and antimicrobial resistance are associated with an increased length of stay and hospital costs, while they have also been linked to high morbidity and mortality rates. In 2016 and 2017, the latest point prevalence survey (PPS) of HAIs and antimicrobial use in European acute care hospitals highlighted an HAI prevalence of 6.5%, while Greece had a higher HAI prevalence of 10%. The aim of this PPS was to record the prevalence of HAIs and antimicrobial use in all eight public acute care hospitals in Crete, Greece during the COVID-19 pandemic in order to highlight the types of infections and antimicrobial practices that need to be prioritized for infection control initiatives. Methods: The PPS was conducted between 30 March and 15 April 2022, according to the ECDC standardized relevant protocol (version 5.3). Statistics were extracted using the ECDC Helics.Win.Net application (software version 4.1.0). Results: A total of 1188 patients were included. The overall point prevalence of patients with at least one HAI was 10.6%. The most frequent types of infections were pneumonia (34.3%), bloodstream infections (10.5%), systemic infections and urinary tract infections (10.5% and 9.1%, respectively). In 14 (12.4%) cases, the pathogen responsible for HAI was SARS-CoV-2 following onsite spread, accounting for almost 10% of all HAIs. Microorganisms were identified in 60.1% of HAIs. Antimicrobials were administered in 711 (59.8%) patients, with 1.59 antimicrobials used per patient. Conclusion: The prevalence of HAI and antimicrobial use among hospitalized patients in Crete, Greece was similar to the national HAI prevalence in 2016 despite the enormous pressure on public hospitals due to the COVID-19 pandemic. Nevertheless, both HAI prevalence and antimicrobial use remain high, underlining the need to implement adequate infection control and antimicrobial stewardship interventions.

## 1. Introduction

Healthcare-associated infections (HAIs) and antimicrobial resistance are both associated with increased length of stay and hospital costs, while they have also been associated with high morbidity and mortality rates [1,2,3]. Surveillance for HAIs at a national level with timely data feedback has been suggested by the World Health Organization as a core component of infection prevention and control in order to reduce HAIs and transmission of antimicrobial resistance [4]. In Greece, national legislation suggests that point prevalence surveys (PPS) of hospital infection and antimicrobial use should be performed every two years [5].

In a previous PPS in 37 acute care hospitals in Greece, the prevalence of HAIs was 9.1%, with an estimated annual HAI incidence of 5.2%, which corresponds to approximately 121,000 patients affected per year [6]. However, in a more recent PPS conducted by the European Centre for Disease Prevention and Control (ECDC), the overall prevalence of HAIs in European acute care hospitals was 6.5%, while data from 42 Greek ones were 10% [7]. This implies that there is potential for improvement by implementing infection control measures to reduce HAIs in Greek acute-care hospitals. To achieve this, evaluation of the current situation by recording HAIs in our hospitals was a requirement.

This need emerged more strongly during the COVID-19 pandemic that has put severe pressure on the healthcare systems not only because of the need to support patients with COVID-19, but also because of the need to avoid the nosocomial spread of COVID-19 that could greatly increase the rates of HAIs. More specifically, COVID-19 could be associated with an increased risk of HAI due to the increased likelihood of COVID-19 transmission, but also due to the higher prevalence of bacterial and fungal HAIs in patients diagnosed with COVID-19 [8]. Importantly, during the COVID-19 pandemic, crowded hospitals, workforce shortage and exhaustion as well as lack of material and equipment became important challenges that should be overcome in order to avoid the spread of COVID-19 in the hospital. To this end, aggressive infection prevention and control measures have been implemented to reduce the nosocomial spread of COVID-19 [9,10].

The aim of this PPS, the first performed during the COVID-19 pandemic, was to record the prevalence of HAIs and antimicrobial use in public hospitals in Crete, Greece in order to highlight the types of infections and antimicrobial practices that need to be prioritized for infection control initiatives. Knowledge of the exact prevalence of HAIs, especially at the time of the COVID-19 pandemic that put high pressure on the healthcare system, could support infection control efforts by highlighting targeted initiatives. This knowledge could also lead to implementation of antimicrobial stewardship measures to reduce unnecessary antimicrobial use and, in the long term, reduce the development of antimicrobial resistance in the healthcare setting.

## 2. Materials and Methods

This PPS was a local initiative of the Infection Control Committee of 7th Health Region of Greece (protocol number 12350/23-3-2022) in accordance with the national legislation (Ministerial Decision Y1.114971/18.02.2014). An invitation for voluntary participation in this PPS was sent to Infection Control Committees of all eight public acute care hospitals of the health region and the acceptance rate was 100%. Thus, this PPS was performed in all eight public hospitals and the sample included all eligible patients hospitalized in these hospitals in Crete, Greece on a single day. Data collection is suggested by Greek legislation that anticipates that PPS of hospital infection and antimicrobial consumption is to be performed every two years [5]; thus, separate approvals by the institutional ethics committees in participating hospitals and patients’ informed consent were not required. All data were anonymous and confidential.

The study was conducted from 30 March 2022 to 15 April 2022 by an expert team of 19 members (infection control nurses, infectious diseases specialists and internal medicine physicians) who were qualified by the relevant course in the European Committee on Infection Control (EUCIC) and who also had participation experience in previous national PPS. The study was conducted according to the ECDC protocol (version 5.3) for point prevalence surveys of HAIs and antimicrobial use that was available at that time [11]. Data regarding total bed capacity and intensive care unit (ICU) beds, number of admissions per year, liters of alcoholic hand rub consumption per year, number of airborne infection isolation rooms, number of infection control nurses and infectious diseases physicians were collected from all participating hospitals.

All patients admitted to a ward before 8:00 am and not discharged from the ward at the time of the survey were included in the study. Patients undergoing same-day medical or surgical treatment, those in the Emergency Department, and outpatients undergoing dialysis were excluded from this study. Data were collected on a single day at the ward/unit level and within a maximum of three days at the hospital level. Data were extracted after review of patients’ nursing and medical hard copy and/or electronic records and on the basis of information that was provided by the nurses and physicians who were in charge of the patients. The collection data form for each eligible patient (including patients not receiving an antimicrobial and not suffering a healthcare-associated infection) included demographics, patient specialty, risk factors (for example, presence of catheters, endotracheal tube and prior surgery), use of antimicrobials and severity of underlying disease, which was determined according to the McCabe classification. Antiviral agents and antimicrobials for the treatment of mycobacteria were excluded. Active HAIs were identified according to ECDC case definitions [11]. Data collected for HAIs included site, date of onset, identified microorganisms and presence of antimicrobial resistance according to data available during the survey. For the data analysis, HAIs were categorized according to the ECDC criteria for all type of infections. All raw data were input into the available ECDC’s website, which was at that time ECDC’s Helics.Win.Net application (software version 4.1.0), which was available at that time (spring 2022), for statistical analysis.

The point prevalence of HAIs was calculated as the rate of patients with at least one HAI divided by the total number of patients. The duration of infection was estimated as the time between the onset of the infection and the date of the survey.

## 3. Results

All eight public acute-care hospitals in Crete, Greece were invited and agreed to participate in this study. The bed size capacity of the participating hospitals ranged from 27 to 771 (median 183.5), while the total bed capacity was 2257. The most common ward types were medical (40, 38.8%), surgical (28, 27.2%), mixed (8, 7.7%), pediatrics (8, 7.7%), ICU (8, 7.7%) (more specifically, general-mixed 5, exclusively for COVID-19 1, pediatric 1, cardiac 1), gynecology and obstetrics (4, 3.9%), psychiatry (4, 3.9%) and neonatology (3, 2.9%). All hospitals, except for one, had at least one full-time infection control nurse; however, only two hospitals had at least one full-time infectious diseases physician. Alcoholic hand rub consumption in total was approximately 318 mL per admission which corresponds to approximately 97 mL per patient-day. Only two hospitals had airborne infection isolation rooms at the time of the survey. Characteristics of the participating hospitals can be seen in Supplementary Table S1.

The data were recorded for 1188 inpatients. The number of patients with HAIs was 126; thus, the prevalence of HAI in this study was 10.6%. The total number of HAIs was 143; therefore, the number of HAIs per patient with at least one HAI was 1.13. The number of HAIs with a specific identified microorganism was 86 (60.1%). HAI was present upon admission (due to recent hospitalization and discharge) in 18.2% (26 cases), with the origin of HAI being the same hospital in 53.8% (14 cases), other hospitals in 38.5% (10 cases) and other or unknown in 7.7% (2 cases). HAI was diagnosed during the current hospitalization in 81.1% (116 cases), and in 0.7% (1 case), data were missing. The HAI was associated with the ward where the survey was conducted in 63.3% (88 cases). Table 1 shows the distribution of HAIs with respect to the type of infection.

Eighty-six patients with HAIs had at least one positive culture, yielding 113 microorganisms in total. The distribution of the microorganisms in the patients with HAI is shown in Table 2.

Table 3 shows data regarding antimicrobial resistance from isolated strains. Antimicrobials were administered in 59.8% (711 of patients) (95% confidence interval 57.1–62.6%), with 1127 antimicrobials being used in total, meaning 1.59 prescribed antimicrobials per patient. Among them, 91% (1026) were administered parenterally, 8.8% (99) were administered orally, and 0.2% (2) via inhalation or rectally. A specific reason for the administration of antimicrobials was documented in the medical records in only 17.9% (202 patients). Antimicrobials were started on day 1 or 2 in 48.5% (547 patients), on day 3 or 4 in 8.8% (99 patients), on days 5–7 in 9% (101 patients), on days 8–14 in 11.9% (134 patients), in days 15–21 in 3% (34 patients), and later on in 10.7% (121 patients), while this information was missing from patients’ records in 8.1% (91 patients). Antimicrobials were used in medical wards in 63.1% of medical patients (388 of 615), in surgical wards in 67.3% of surgical patients (214 of 318), in ICU in 69.9% of such patients (58 of 83), in obstetrics and gynecology in 49.1% of such patients (28 of 57), in pediatrics in 34.3% of pediatric patients (12 of 35) and in psychiatric wards in 6 such patients (8.5% of 71).

The indications for antimicrobial use were treatment in 40.9% (486 patients), and more specifically, community-acquired infection in 30.1% (357 patients), HAI in 10.4% (124) and long-term-care-associated HAI in 0.5% (6 patients). Surgical prophylaxis was the indication for antimicrobial use in 12.2% (145 patients), with antimicrobial use being single-dose administration in 2.1% (25 patients), one-day administration in 1.3% (16 patients) and longer than one day in 8.9% (106 patients). Medical prophylaxis was the indication for administration in 6.6% (79 patients), while other indications were noted in 0.8% (9 patients), and indications were unknown in 0.9% (11 patients). Infections associated with antimicrobial use were respiratory tract infection in 22.5% (254 cases), being acute bronchitis or exacerbation of chronic bronchitis in 7% (79 cases), and pneumonia in 15.5% (175 cases); UTI in 4.3% (49 cases), being symptomatic lower UTI in 2.7% (30 cases), symptomatic upper UTI in 1.6% (18 cases), and asymptomatic bacteriuria in 0.1% (1 case); systemic infection in 5.7% (64 cases), being laboratory-confirmed bacteremia in 2.2% (25 cases), clinical sepsis excluding febrile neutropenia in 2.6% (29 cases), systemic inflammatory response with no clear anatomic site in 0.8% (9 cases) and complete undefined site with no systemic inflammation in 0.1% (1 case); cardiovascular infection in 0.7% (8 cases); gastro-intestinal infection in 6.6% (74 cases), with 3.9% (44 cases) of them being intraabdominal sepsis including hepatobiliary infections; skin and soft tissue surgical site infections in 1.3% (15 cases); bone and joint surgical site infections in 0.2% (2 cases); other skin and soft tissue infections in 1.4% (16 cases); other bone and joint infections in 0.4% (5 cases); central nervous system infections in 0.4% (5 cases); infections of ear, mouth, nose, throat or larynx in 1.4% (16 cases); obstetric or gynecological infections or sexually transmitted diseases (STDs) in women in 0.3% (3 cases), prostatitis, epididymitis/orchitis and STDs in men in 0.4% (4 cases). Indication was missing/unknown in 0.4% (4 cases). Supplementary Table S2 shows the type of antimicrobials used.

## 4. Discussion

This is the first multicenter PPS in Crete, Greece which aimed to identify the prevalence of HAIs and antimicrobial consumption in all hospitals in the region, and more specifically, in the era of the COVID-19 pandemic. Importantly, we identified that more than one out of ten hospitalized patients in the Cretan hospitals suffer from HAI, with 10% of them being due to COVID-19. The most common HAIs were pneumonia, bloodstream infections and systemic infections, followed by UTIs and surgical site infections.

The prevalence of HAIs in this study was higher than that noted in another recent Greek study that was estimated at 9.1% [6]. Furthermore, the prevalence of HAIs in the present study was even higher than the prevalence noted by the ECDC, which was estimated at 5% [12]. In a more recent European study from 2016 to 2017, a PPS of HAIs and antimicrobial use in European acute care hospitals highlighted a HAI prevalence of 6.5%, while Greece had a higher HAI prevalence of 10%, a prevalence slightly lower than that identified in the present study [7]. The prevalence of HAI in the present study was also higher than that in other countries before the pandemic, such as in Australia, Switzerland, Scotland and Japan, where the prevalence of HAI was 9.9%, 5.4%, 4.6% and 10.1%, respectively [3,13,14,15], but lower than that in another study performed in Singapore, where it was 11.9% [16]. The same ECDC definitions were used in all the above-mentioned studies. In another study performed in the USA, the prevalence of HAI was 4%, whereas in a study from Canada, the prevalence was 10.5%, although different definitions were used in those studies [1,17,18]. These differences noted among different countries may be due to real differences in HAI prevalence but may also reflect differences in healthcare infrastructure and healthcare services or differences in the design of those studies. However, it is of critical importance to note that the present study was the only one performed during the COVID-19 pandemic, while approximately 10% of HAIs in the present PPS were due to COVID-19, implying that the actual HAI prevalence herein, excluding COVID-19, would be at most 9.6%, a number similar to that in a previous PPS in Greece [6]. Thus, caution is needed when comparing results from PPS performed in different countries and under different circumstances.

In the studies conducted in Greece, the prevalence of HAI was slightly lower than that noted herein [6,7]. This could be attributed to the fact that the present study was performed after the onset of the COVID-19 pandemic, which has seriously affected several aspects of antimicrobial resistance, antimicrobial stewardship and infection control, thus leading to higher rates of HAIs [9,19,20,21,22].

In the present study, the most common HAIs were pneumonia and bloodstream infection, while in other studies, as well as in the study from the ECDC, surgical site infections were the most common, followed by pneumonia [12,13]. This difference could be due to local factors associated with the number of surgeries performed per hospital or with the reduced number of surgical interventions associated with restrictions due to the COVID-19 pandemic. This may also represent an increased likelihood of hospital-acquired pneumonia and bloodstream infections in the hospitals surveyed in the present study. This could allow for improvement since implementation of infection control measures through application of specific bundles of care could lead to reduced rates of pneumonia and bloodstream infections [23,24]. Notably, in a previous Greek study, lower respiratory tract infections were the most common HAIs, followed by bloodstream infections, which is in line with the findings of the present study [6].

Interestingly, the most commonly identified microorganism was *Acinetobacter baumannii*, which in the vast majority of cases was extensively drug-resistant (XDR). Antimicrobial resistance was also very high for other commonly isolated microorganisms, such as *Enterobacterales*, *Pseudomonas* spp., staphylococci and enterococci, as was the case in a previous PPS from Greek hospitals [6]. Indeed, there is an alarmingly increasing antimicrobial resistance noted in Greek hospitals and the community, while, on the other hand, the high prevalence of antimicrobial resistance noted can probably be correlated with the high consumption of antimicrobials. There are several studies directly linking the use of antimicrobials with the development of antimicrobial resistance [25,26,27,28,29]. More than 50% of patients in this survey were receiving antimicrobials. In another recent study, again, the majority of the patients from Greek hospitals (56%) were also noted to be receiving antimicrobials, while the corresponding average rate for all European countries was approximately 33% [30]. This indicates a high need for the implementation of antimicrobial stewardship measures to reduce unnecessary antimicrobial use and, in the long term, reduce the development of antimicrobial resistance. For example, a significant proportion of patients who received antimicrobials for surgical prophylaxis in the present study received antimicrobials for more than one day. Furthermore, the empowerment of appropriate infection control measures to reduce in-hospital transmission of resistant microorganisms is critical [31].

The findings of the present study should be studied with an adequate understanding of the present situation of infection control and antimicrobial stewardship practices in Crete. National legislation in Greece mandates the provision of infection control nursing service in every hospital (Ministerial Decision Y1/4234/2001–F.E.K. 733 B’ 12-07-2001). All participating hospitals herein, with only one single exception (a regional hospital in rural Crete), had at least one full-time infection control nurse, even though that one hospital did have an infection control nurse who was not on full-time duty. The significance of the role of full-time infection control nursing service in the reduction of the spread of antimicrobial resistance and hospital infection in the hospital has been recently proven [32,33]. Before the emergence of the COVID-19 pandemic, literature suggested at least one dedicated infection control nurse per 100 beds in acute-care centers [33,34,35,36,37,38]. After the emergence of the COVID-19 pandemic, however, consultation requests in infection prevention and control programs noted an enormous increase that was up to 500%, thus increasing work stress for infection control nurses and possibly undermining the quality of their work [39].

Regarding application of infection control measures in the Greek hospitals, a recent survey in Greek hospitals revealed that, on average, the surveyed hospitals were at an intermediate level in terms of hand hygiene practice, while there was considerable room for improvement in staffing levels and resources. On the other hand, there was room for improvement in terms of hand hygiene practices, by setting specific indicators for healthcare workers and stronger patient involvement [40]. In the present study, alcoholic hand rub consumption was 97 mL per patient-day, which was above the suggested limit for daily use, even though the present survey was performed during the COVID-19 pandemic; thus, alcoholic hand rub consumption may have greatly changed, and previous targets may not reflect current needs [41,42].

Even though full-time infection control nurses and full-time infectious disease physicians were not employed in all participating hospitals, the region of Crete employs an infection control network consisting of both infectious diseases physicians and infection control nurses for providing consultative service to all hospitals that may request assistance. Infection control networks have been shown to effectively reduce the incidence of HAIs in other countries [43,44]. To that end, application of surveillance methods, data analysis and feedback on a regular basis, and guideline-based interventions according to the ECDC and national infection control guidelines could be effectively employed through this outreach program to lessen the rate of HAIs. To that end, a future perspective could be to further strengthen the outreach program and network between the infection control service of the different hospitals, organize educational activities to increase awareness of antimicrobial stewardship and evaluate the effects of these interventions through conduction of future PPS in the collaborating hospitals.

This study has some limitations that should be noted. First of all, it was conducted in a small number of hospitals in a geographically restricted area during the COVID-19 pandemic. Generalization of the results to the rest of Greece should be performed with caution. Furthermore, discrepancies among the participating hospitals may make the unification of the results problematic. For example, even though there were many instances of microbiological data in the present study, they were mostly derived from the four largest hospitals that participated in this study. Finally, although there are some data on antimicrobial resistance, this was not the primary aim of this study; therefore, data on this topic may be limited.

## 5. Conclusions

This is the first PPS performed after the emergence of the COVID-19 pandemic, providing data on the prevalence of HAIs and the antimicrobial use in Greek hospitals, and more specifically, on the island of Crete. A high prevalence of HAIs and antimicrobial use was noted, which underscores the high need for implementation of adequate infection control and antimicrobial stewardship practices. More specifically, the information provided about the exact prevalence of HAIs, the antimicrobial use and, to some extent, about antimicrobial resistance during the time of the COVID-19 pandemic by this study could be used to improve everyday practice. To that end, actions could be taken regarding intensification of education of healthcare personnel regarding infection control and antimicrobial stewardship practices, while conduction of PPS in the future could be used as measures to estimate it.

## Figures and Tables

**Table 1:** Distribution of hospital-acquired infections in regards to the site of infection.

HAI Type	Number of HAI	Percent of HAIs
Pneumonia	49	34.3%
Bloodstream infections	22	15.4%
Systemic infections *	15	10.5%
Urinary tract infections	13	9.1%
Surgical site infections	12	8.4%
Eye, ear, nose or mouth infection	11	7.7%
Another lower respiratory tract infection	8	5.6%
Gastrointestinal system infections	6	4.2%
Cardiovascular infections	2	1.4%
Skin and soft tissue infections	2	1.4%
Catheter-related infections without bloodstream infection	1	0.7%
Bone and joint infections	1	0.7%
Reproductive tract infections	1	0.7%
Total	143	100%

HAI: hospital-acquired infection. * Treated unidentified severe infection in adults and children and systemic infections, category not specified/unknown.

**Table 2:** Microorganism distribution in patients with hospital-acquired infection.

Microorganism	Number of Patients	Percentage
Gram-negatives	53	46.9%
*Acinetobacter baumannii*	24	21.2%
*Escherichia coli*	5	4.4%
*Hafnia* spp.	2	1.8%
*Morganella* spp.	1	0.9%
*Klebsiella pneumoniae*	7	6.2%
*Proteus mirabilis*	3	2.7%
*Pseudomonas aeruginosa*	9	8%
*Alcaligenes* spp.	1	0.9%
*Stenotrophomonas maltophilia*	1	0.9%
Gram-positive cocci	28	24.8%
*Enterococcus faecalis*	2	1.8%
*Enterococcus faecium*	10	8.8%
Other *Enterococcus* spp.	1	0.9%
*Staphylococcus aureus*	7	6.2%
*Staphylococcus epidermidis*	6	5.3%
*Streptococcus* spp.	2	1.8%
SARS-CoV-2	14	12.4%
Fungi	13	11.5%
*Aspergillus* spp.	1	0.9%
*Candida* spp.	12	10.6%
Anaerobic bacilli	3	2.7%
*Clostridium difficile*	3	2.7%
Gram-positive bacilli	2	1.8%
*Bacillus* spp.	1	0.9%
*Corynebacterium* spp.	1	0.9%
Total	113	100%

SARS-CoV-2: severe acute respiratory syndrome coronavirus 2.

**Table 3:** Antimicrobial resistance data among strains with available data isolated from patients with hospital-acquired infection.

Microorganism/Resistance	Isolates Tested
MRSA	3 out of 6 *Staphylococcus aureus* tested (50%)
VRE	6 out of 13 *Enterococci* tested (46.2%)
Enterobacteriaceae resistant to 3rd generation cephalosporins	5 out of 12 strains tested (41.7%)
Enterobacteriaceae resistant to carbapenems	3 out of 13 strains tested (23.1%)
*Pseudomonas aeruginosa* resistant to carbapenems	3 out of 7 strains tested (42.9%)
*Acinetobacter baumannii* resistant to carbapenems	23 out of 23 strains tested (100%)

MRSA: methicillin-resistant *Staphylococcus aureus*; VRE: vancomycin-resistant *Enterococcus* spp.

## Data Availability

The data presented in this study are available on request from the corresponding authors.

## Supplementary Materials

The following supporting information can be downloaded at: https://www.mdpi.com/article/10.3390/antibiotics11091258/s1, Table S1: Characteristics of the participating hospitals; Table S2: Type of antimicrobials used.
