# Supplementary material for: A Point Prevalence Survey of Healthcare-Associated Infections and Antimicrobial Use in Public Acute Care Hospitals in Crete, Greece
Source: Antibiotics (Basel). 2022 Sep 16;11(9):1258. doi: 10.3390/antibiotics11091258 (PMC9495163; doi:10.3390/antibiotics11091258)
Supplement: Supplementary file 1 [file antibiotics-11-01258-s001.zip › antibiotics-1899711-supplementary.pdf]

Table S1. Characteristics of the participating hospitals.

Hospital code	701	702	703	704	705	706	707	708	Total
**Hospital beds in total, n**	147	440	75	27	75	220	502	771	2,257
**ICU beds, n**	6	15	0	0	0	7	12	67	107
**Patients included in PPS, n**	63	233	28	12	33	104	272	443	1188
**Admissions per year *, n**	8192	36,525	3295	415	1,751	9,383	30,568	61,168	151,297
**Alcoholic hand rub consumption, liters/year**	3200	10,415	875	307	1,236	3,087	11,489	17,453	48,062
**Full-time infection control nurses, n**	1	1	0	1	1	1	2	4	11
**Full time infectious diseases physicians, n**	0	0	0	0	0	1	0	4	5
**Airborne infection isolation rooms, n**	0	0	0	0	0	6	0	7	13

ICU: intensive care unit; PPS: point-prevalence survey; * Data refer to 2020 or 2021.

Table S2. Type of antimicrobials used.

Antimicrobial	Total	%	Treatment	%	SP	%	MP	%	Other	%
Colistin oral	1	0.1	1	0.1	0	0	0	0	0	0
Rifaximin	1	0.1	0	0	0	0	1	0.9	0	0
Vancomycin oral	8	0.7	6	0.7	0	0	2	1.9	0	0
Doxycycline	15	1.3	12	1.5	0	0	3	2.8	0	0
Tigecycline	35	3.1	33	4.1	2	1.1	0	0	0	0
Amoxicillin	6	0.5	4	0.5	1	0.5	1	0.9	0	0
Ampicillin	9	0.8	5	0.6	1	0.5	2	1.9	1	3.7
Piperacillin	11	1	10	1.2	0	0	1	0.9	0	0
Benzylpenicillin	1	0.1	1	0.1	0	0	0	0	0	0
Cloxacillin	3	0.3	3	0.4	0	0	0	0	0	0
Amoxicillin and inhibitor	7	0.6	4	0.5	2	1.1	1	0.9	0	0
Ampicillin and inhibitor	43	3.8	34	4.2	4	2.1	1	0.9	4	14.8
Piperacillin and inhibitor	106	9.4	81	10.1	8	4.2	11	10.2	6	22.2
Sultamicillin	3	0.3	1	0.1	1	0.5	1	0.9	0	0
Second generation cephalosporins	118	10.5	25	3.1	80	42.1	10	9.3	3	11.1
Cefaclor	1	0.1	0	0	0	0	1	0.9	0	0
Ceforanide	15	1.3	1	0.1	12	6.3	0	0	2	7.4
Cefoxitin	64	5.7	14	1.7	45	23.7	4	3.7	1	3.7
Cefprozil	6	0.5	0	0	6	3.2	0	0	0	0
Cefuroxime	32	2.8	10	1.2	17	8.9	5	4.6	0	0
Third generation cephalosporins	124	11	110	13.7	9	4.7	5	4.6	0	0
Cefixime	1	0.1	1	0.1	0	0	0	0	0	0
Cefotaxime	9	0.8	9	1.1	0	0	0	0	0	0
Ceftazidime	11	1	7	0.9	2	1.1	2	1.9	0	0
Ceftriaxone	97	8.6	87	10.8	7	3.7	3	2.8	0	0
Ceftazidime combinations	6	0.5	6	0.7	0	0	0	0	0	0
Fourth generation cephalosporin (cefepime)	13	1.2	11	1.4	0	0	2	1.9	0	0
Carbapenems	81	7.2	72	9	2	1.1	5	4.6	2	7.4
Ertapenem	1	0.1	1	0.1	0	0	0	0	0	0
Imipenem and inhibitor	2	0.2	2	0.2	0	0	0	0	0	0
Meropenem	78	6.9	69	8.6	2	1.1	5	4.6	2	7.4
Ceftaroline	5	0.4	5	0.6	0	0	0	0	0	0
Ceftolozane and inhibitor	2	0.2	2	0.2	0	0	0	0	0	0
Sulfamethoxazole and trimethoprim	20	1.8	5	0.6	0	0	15	13.9	0	0
Sulfametrole and trimethoprim	1	0.1	0	0	0	0	1	0.9	0	0
Macrolides	28	2.5	25	3.1	1	0.5	1	0.9	1	3.7
Azithromycin	24	2.1	22	2.7	0	0	1	0.9	1	3.7
Clarithromycin	3	0.3	2	0.2	1	0.5	0	0	0	0
Erythromycin	1	0.1	1	0.1	0	0	0	0	0	0
Clindamycin	35	3.1	24	3	10	5.3	1	0.9	0	0
Aminoglycosides	25	2.2	16	2	6	3.2	1	0.9	2	7.4
Amikacin	18	1.6	11	1.4	6	3.2	1	0.9	1	3.7
Gentamicin	7	0.6	5	0.6	0	0	1	0.9	1	3.7
Fluoroquinolones	127	11.3	96	12	17	8.9	13	12	1	3.7
Ciprofloxacin	58	5.1	36	4.5	16	8.4	6	5.6	0	0

Levofloxacin	40	3.5	32	4	1	0.5	6	5.6	1	3.7
Moxifloxacin	29	2.6	28	3.5	0	0	1	0.9	0	0
Glycopeptides	99	8.8	59	7.4	29	15.3	10	9.3	1	3.7
Teicoplanin	56	5	28	3.5	22	11.6	5	4.6	1	3.7
Vancomycin parenteral	43	3.8	31	3.7	7	3.7	5	4.6	0	0
Colistin	29	2.6	28	3.5	0	0	0	0	1	3.7
Metronidazole parenteral	68	6	49	6.1	13	6.8	6	5.6	0	0
Daptomycin	21	1.9	19	2.4	0	0	2	1.9	0	0
Linezolid	23	2	21	2.6	0	0	1	0.9	1	3.7
Amphotericin B parenteral	4	0.4	1	0.1	0	0	3	2.8	0	0
Miconazole	1	0.1	1	0.1	0	0	0	0	0	0
Triazole derivatives	20	1.8	13	1.6	2	1.1	4	3.7	1	3.7
Fluconazole	14	1.2	10	1.2	2	1.1	2	1.9	0	0
Posaconazole	1	0.1	0	0	0	0	1	0.9	0	0
Voriconazole	2	0.2	2	0.2	0	0	0	0	0	0
Isavuconazole	3	0.3	1	0.1	0	0	1	0.9	1	3.7
Anidulafungin	6	0.5	6	0.7	0	0	0	0	0	0
Micafungin	16	1.4	12	1.5	0	0	3	2.8	1	3.7
Rifampicin	5	0.4	3	0.4	0	0	1	0.9	1	3.7
Metronidazole oral/rectal	7	0.6	4	0.5	2	1.1	0	0	1	3.7
Total antimicrobials	1127	100	802	100	190	100	108	100	27	100

MP: medical prophylaxis; SP: surgical prophylaxis
